# Measures of neck muscle strength and their measurement properties in adults with chronic neck pain—a systematic review

**DOI:** 10.1186/s13643-022-02162-5

**Published:** 2023-01-16

**Authors:** Deepa Abichandani, Jonathan Tong Yuk Ting, Edith Elgueta Cancino, Shouq Althobaiti, Deborah Falla

**Affiliations:** 1 Department of Physiotherapy, Institute of Health and Social Care, London South Bank University, London, UK; 2 Centre of Precision Rehabilitation for Spinal Pain (CPR Spine), School of Sport, Exercise and Rehabilitation Sciences, University of Birmingham, Birmingham, UK

## Abstract

**Background:**

Measurement of neck muscle strength is common during the assessment of people with chronic neck pain (CNP). This systematic review evaluates the measurement properties (reliability, validity, and responsiveness) of neck muscle strength measures in people with CNP.

**Databases and data treatment:**

This systematic review followed a PROSPERO registered protocol (CRD42021233290). Electronic databases MEDLINE (OVID interface), CINAHL, SPORTDiscuss via (EBSCO interface), EMBASE (OVID interface), and Web of Science were searched from inception to 21 June 2021. Screening, data extraction, and quality assessment (Consensus-based Standards for the selection of Health Measurement Instruments (COSMIN) checklist) were conducted independently by two reviewers. The overall strength of evidence was evaluated using the modified Grading of Recommendations Assessment, Development and Evaluation.

**Results:**

From 794 records, nine articles were included in this review which concerned six different neck strength outcome measures. All studies evaluated reliability and one evaluated construct validity. The reliability of neck strength measures ranged from good to excellent. However, the risk of bias was rated as doubtful/inadequate for all except one study and the overall certainty of evidence was rated low/very low for all measures except for the measurement error of a handheld dynamometer.

**Conclusion:**

A multitude of measures are used to evaluate neck muscle strength in people with CNP, but their measurement properties have not been fully established. Further methodologically rigorous research is required to increase the overall quality of evidence.

**Supplementary Information:**

The online version contains supplementary material available at 10.1186/s13643-022-02162-5.

## Introduction

Musculoskeletal disorders are ranked second in contributing years lived with disability worldwide [34]. Within the spectrum of musculoskeletal disorders, neck pain is a very common condition with a high age-standardized lifetime prevalence of 66.7% [5] and 12-month prevalence rates varying from 20 to 40% [9].

People with neck pain commonly present with altered physical function including neck muscle weakness [14, 15, 37]. Neck muscle strength training is known to be an effective intervention for patients with neck pain [1, 8], and an association exists between the extent of the reduction in neck pain and disability and an increase in neck strength following neck strengthening in people with chronic neck pain (CNP) [36]. The measurement of neck strength is therefore relevant to determine the presence of neck muscle weakness and to monitor strength changes over time as it serves as an important objective marker throughout the course of rehabilitation as are other objective markers [16].

Numerous methods have been used to evaluate neck strength, including manual muscle testing [24], hand-held dynamometry [32], strain-gauge dynamometry [12], isometric [35], and isokinetic tests [2] and specialized equipment such as the multi cervical unit [4]. It is imperative that clinicians are utilizing performance-based outcome measures (PBOM) that meet certain benchmarks for measurement properties to ensure the highest clinical accuracy [7]; the COSMIN initiative (Consensus-based Standards for the selection of Health Management Instruments) have standardized the terminologies and taxonomy of relevant measurement properties for instrument evaluation under a consensus-based approach [21–23], which are reliability, validity, and responsiveness.

A systematic review conducted by de Koning et al. [6] evaluated clinimetric properties of tests of neck muscle functioning in patients with neck pain. However, it primarily focused on the measurement properties of measures for neck muscle endurance. The review highlighted the lack of portable neck strength assessment tools that can examine neck strength in a reliable manner. More recently, Selistre et al. [29] conducted a systematic review exploring clinical tests utilized to measure neck muscle strength or endurance in participants with non-specific CNP or asymptomatic participants. However, the authors only included tests that could be performed within a maximum of 5 min and involved equipment with a maximum cost of €1000, which limited the number of tests considered. Thus, the review was not able to provide an overview of all methods currently tested for their measurement properties for the assessment of neck strength in people with CNP; it is relevant to understand how the measurement properties (e.g., reliability) of low-cost approaches compare to those of a ‘gold-standard’ (i.e., isokinetic dynamometer). Additionally, the Grading of Recommendations Assessment, Development, and Evaluation (GRADE) approach was not adopted to examine the overall quality of evidence regarding the measurement properties, yet this is an important process to appreciate the trustworthiness of summarized results.

Thus, in the current systematic review, we aimed to appraise the psychometric properties of various neck strength outcome measures (without limits on the duration of testing or cost of the equipment) and establish their appropriateness for the evaluation of neck strength in patients with chronic neck pain based on their measurement properties. This rigorous systematic review applied the COSMIN Risk of Bias checklist, and the study results were rated against the COSMIN criteria for good measurement properties. Additionally, the GRADE approach was used to draw conclusions on the overall strength of the evidence.

## Methodology

The reporting of this systematic review adheres to the Preferred Reporting Items for Systematic Reviews and Meta-Analysis (PRISMA) checklist [13]. The review was designed based on the COnsensus-based Standards for the selection of health Measurement INstruments COSMIN methodology [19–23]. A registered summary of this protocol is available on PROSPERO (CRD42021233290).

### Eligibility criteria

#### Inclusion criteria

For studies to be included in this systematic review, they were required to meet the following eligibility criteria: *(1) Target population*: studies with adult participants (>18 years), who are experiencing CNP of either non-traumatic or traumatic origin; *(2) Outcome measure*: studies investigating PBOM of neck strength (manual, mechanical, and functional techniques); *(3) setting*: studies that evaluate the measurement properties of PBOM of neck strength in a laboratory, clinical, or field-based environment; and *(4) Measurement properties*: studies that evaluate one or more clinimetric properties of PBOM based on the COSMIN taxonomy (e.g., reliability, validity, and responsiveness) [21–23].

#### Exclusion criteria

Studies were excluded according to the following criteria: *(1) Language*: studies published in a language other than English due to restricted ability in language translation. *(2) Article type*: studies that were either conference abstracts, articles without full-text availability or systematic review articles; and *(3) Study demographic*: the study only evaluated asymptomatic participants.

### Information sources and search strategy

A comprehensive literature search was conducted using medical subject headings and free text, and relevant keywords were identified during scoping searches. MEDLINE (OVID interface), CINAHL, SPORTDiscuss via (EBSCO interface), EMBASE (OVID interface), and Web of Science were electronically searched from inception until 21 June 2021 to maximize literature coverage, as per Cochrane collaboration recommendations [10]. To identify additional literature, a hand searching of reference lists of relevant articles was conducted. Gray literature and conference papers were searched to reduce potential publication biases.

The search strategy was established with the MEDLINE database, and changes and adaptations were made when undergoing search processes in other databases. The search strategy used in MEDLINE (OVID interface) is reported in Additional file 1: Appendix 1. Specific search terms included keywords and Medical Subject Headings (MeSH) terms related to the neck region, muscle strength, and psychometric properties, e.g., reliability, validity, and responsiveness. Terms describing demographics of interest were also included. In addition, relevant search filters constructed by COSMIN for the purpose of identifying appropriate studies on measurement properties were used [27].

### Study selection

The first reviewer [JT] performed an extensive electronic search on the aforementioned databases. All search results were recorded and exported to EndNote Version X9 (Clarivate analytics) software for abstract and full-text storage. This enabled duplicated studies to be recognized and removed from the software.

**Fig. 1:**
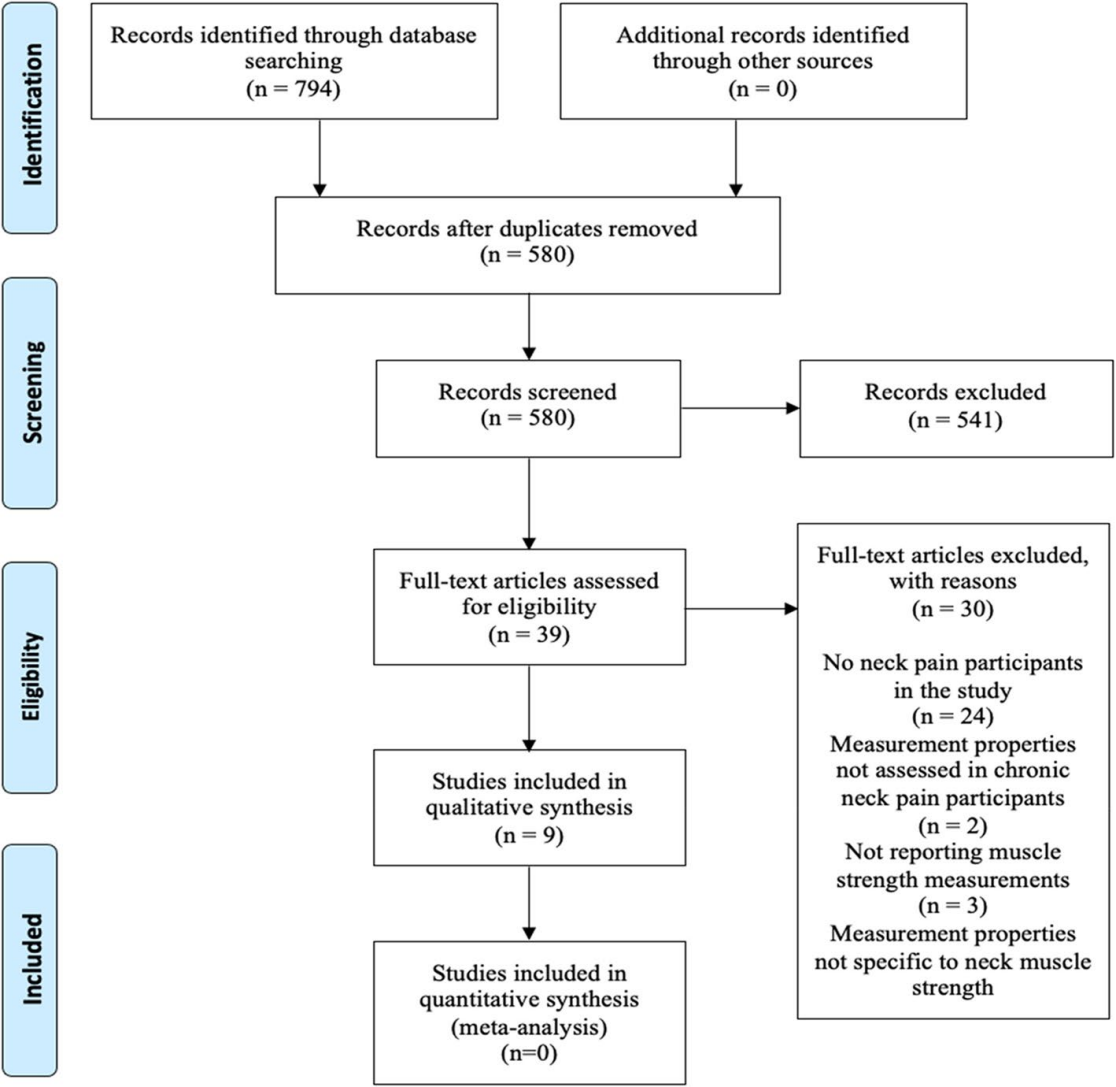
PRISMA flow diagram summarzing the number of articles included at each stage of the review

Based on the eligibility criteria established, two reviewers [JT, DA] independently screened study titles and abstract and designated studies into three subcategories namely “include,” “exclude,” and “unsure” [17]. In addition, each reviewer independently read the full texts that were categorised as “unsure” and assessed against the eligibility criteria [10]. The authors were contacted via email if additional information was needed. Any disagreement regarding study eligibility was resolved either by consensus or involvement of the third reviewer [DF]. The rationale for the exclusion of studies is reported in Fig. 1.

### Data collection process and data items

A standardized form was used to extract relevant data from each included study. Piloting the data collection form was carried out to ensure the collection of all relevant information. Both reviewers [JT, DA] independently utilised the standardized form to extract relevant data. The third reviewer [DF] was available to discuss about any potential disagreements regarding extracted data if needed until concurrence was established. Additional file 1: Appendix 2 outlines the extracted from utilised for the included studies.

### Risk of bias in individual studies

The COSMIN Risk of Bias checklist was implemented to assess the risk of bias in included studies with the utilization of the original COSMIN tool that demonstrates a high level of inter-rater agreement [18–23]. It comprises of standards for design requirements and preferred statistical methods of studies on measurement properties, with ten COSMIN boxes encapsulating benchmarks for PROM development and for nine aspects of measurement properties (reliability, validity, and responsiveness) [27]. The two reviewers individually rated each outcome measure as either very good, adequate, doubtful, or inadequate quality [27]. Disagreements were resolved between the reviewers, and the third reviewer was available to intervene if required for reaching consensus.

### Data synthesis

The characteristics of the included studies in this review were found to be heterogenous in nature (study demographic, methodological design, outcome measures, and statistical design). As a result, it was not possible to be carry out a meta-analysis, and a narrative synthesis was conducted instead. The narrative synthesis was completed in accordance with the COSMIN guidelines for systematic reviews [27]. Results of the included studies per measurement property, per outcome measure, and per test direction were quantitatively pooled and evaluated against the COSMIN criteria for good measurement properties to establish whether the measurement property was sufficient (+), insufficient (−), inconsistent (±), or indeterminate (?) [27].

### Quality of the evidence

A modified GRADE approach was adopted to examine the quality of evidence and the trustworthiness of summarized results [19, 20]. The grading of the quality of evidence was listed as high, moderate, low, or very low evidence. Following the COSMIN recommendation, four determinants of quality of evidence were used: (1) risk of bias (methodological quality of the studies), (2) inconsistency (unexplained inconsistency of results across studies), (3) imprecision (total sample size of the available studies), and (4) indirectness (evidence from different populations than the population of interest in the review). The fifth factor on the GRADE approach, publication bias, was not taken into account due to the lack of registries for studies on measurement properties [19, 20].

## Results

### Study selection

Figure 1 summarizes the articles included at each stage of the review. A total of 794 articles were identified following searches on electronic databases. After duplicate studies were removed, 580 articles were screened at title and abstract stage, with 39 assessed at full-text stage. Finally, a total of 9 studies were included in this review.

### Study characteristics

Tables 1 and 2 present the study characteristics and results of the included 9 studies. One study [26] specifically investigated a population with Whiplash-Associated Disorder (WAD). The remaining 8 studies carried out investigations on chronic neck pain including one study which had a mixed patient group of WAD and non-specific chronic neck pain. All studies investigated reliability [3, 4, 11, 25, 26, 28, 30, 33, 37], one investigated validity [4], but no studies evaluated responsiveness. Neck strength measures evaluated were a handheld dynamometer (HHD) [3, 30], isometric dynamometer [25], strain gauge dynamometer (SGD) [11, 37], modified sphygmomanometer dynamometer (MSD) [33], multi-cervical unit (MCU) [4, 26], and multifunctional measurement unit [28]. The measurement procedures for the individual studies are presented in Additional file 1: Appendix 3.

**Table 1:** Study characteristics of included studies

Study	Country	Study design	Sample size	Participant characteristics	Cause of neck pain	Neck pain characteristics	Outcome measure
Chiu and Lo [4]	Hong Kong	Cross-sectional	Total (*n* = 46) Neck pain (*n* = 21)	Age: Mean (SD) (years) Neck pain: 27.0 (9.5) Gender: M/F All participants: 24:22 Neck pain: 9:12	Mechanical neck pain	**Disability**: Not reported **Pain duration**: Not reported **Intensity of pain**: Not reported	Multi Cervical Rehabilitation (Hanoun Medical Inc., Ontario)
Cibulka et al. [3]	USA	Cross-sectional	Total (*n* = 51) Neck pain (*n* = 37)	Age: Mean (SD) (years) Neck pain: 22.8 (3.5) Gender: M/F All participants: 18:33 Neck pain: 14:23	Mechanical neck pain	**Disability**: Not reported **Pain duration**: Not reported **Intensity of pain**: Not reported	Handheld dynamometer (Microfet) (Hogan Health Industries, Salt Lake City, UT)
Jordan et al. [11]	Denmark	Not reported	Total (*n* = 199) Neck pain (*n* = 119)	Age: Mean (SD) (years) Not reported Gender: M/F All participants: 71:128 Neck pain: 31:88	Not reported	**Disability**: Not reported **Pain duration:** 42 patients reported NP <3 years 40 patients reported NP >3 years 28 patients reported NP >5 years 9 patients reported NP >10 years **Intensity of pain**: Median (IQR) 13 (8.30)	Strain gauge Dynamometer (Neck Exercise Unit, Follo, Norway)
O’Leary et al. [25]	Australia	Not reported	Total (*n* = 20) History of neck pain (*n* = 12)	Age: Mean (range) (years) Total: 27.9 (18–47) Gender: M/F All participants: 5:15	Not reported	**Disability:** Mean (range) History of neck pain 20.4 (10–46) **Pain duration**: Not reported **Intensity of pain**: Not reported	Isometric Dynamometer (NeckMetrix, UniQuest Pty Ltd, The University of Queensland, Brisbane, Australia)
Pearson et al. [26]	Canada	Cross-sectional, repeated-measures design	Total (*n* = 42) Chronic WAD (*n* = 14)	Age: Mean ± SD (range) (years) WAD: 36.6 ± 10.8 (24–58) Gender: M/F All participants: 24:18 WAD: 8:6	Whiplash	**Disability** (NDI) (/50): WAD (/50) 20.0 ± 6.9 (10–33) **Pain duration**: Not reported **Intensity of pain at rest** (/100mm): Session 1–33.4 ± 18.8 Session 2–27.4 ± 20.7	Multi-Cervical Unit (MCU, BTE Technologies, Inc.)
Scheuer and Friedrich [28]	USA	Non-randomized controlled trial	Total (*n* = 95) Neck pain (*n* = 53)	Age: Mean (SD) (years) Neck pain: 49.72 (10.74) Gender: M/F All participants: 26:69 Neck pain: 14:39	Not reported	**Disability**: Not reported **Pain duration**: Not reported **Intensity of pain**: Not reported	(Back Check 607) Multifunctional Measurement Unit (Dr. Wolff Sports & Prevention GmbH, Arnsberg, Germany)
Shahidi et al. [30]	USA	Repeated measures design	Total (*n* = 39) Neck pain (*n* = 19)	Age: Mean (SD) (years) Neck pain: 34.9 (9.9) Gender: M/F All participants: 20:19 Neck pain: 10:9	Not reported	**Disability:** NDI (points): mean (SD) Neck pain 14.4 (7.3) **Pain duration**: Not reported **Intensity of pain**: Not reported	Handheld dynamometer (FPIX 100kg load cell, Wagner Instruments, Greenwich, CT)
Vernon et al. [33]	Canada	Analytic survey	Neck pain (*n* = 24)	Age: Mean (SD) (years) Neck pain: Male: 39 (7) Female: 36 (10) Gender: M/F Neck pain: 12:12	**Mechanical neck pain syndrome** 1. Whiplash-type cervical sprain/strain injury (*n* = 8) 2. Non-traumatic mechanical complaints related to postural or occupational strains (*n* = 16)	**Disability**: Not reported **Pain duration**: Mean 1. Whiplash-type cervical sprain/strain injury: 22.5 weeks 2. Nontraumatic mechanical complaints related to postural or occupational strains: 110 weeks **Intensity of pain**: Not reported	Modified sphygmomanometer dynamometer (Magnatec Co. Ltd., Concord, Ontario, Canada)
Ylinen et al. [37]	Finland	Cross sectional	Total (*n* = 42) Neck pain (*n* = 21)	Age: Mean (SD) (years) Neck pain: 44 (6) Gender: M/F No male 42 females	Non-specific neck pain	**Disability**: Mean ± SD Vernon disability index: 13 ± 5 **Pain duration**: Mean (SD) 9 (6) years **Intensity of pain**: (VAS) 54 ± 22mm	Strain gauge dynamometer (Kuntovaline Inc., Helsinki, Finland)

Key: *NP* neck pain, *NDI* Neck Disability Index, *SD* standard deviation, *WAD* whiplash-associated disorders

**Table 2:** Summary of results of the included studies

Study	Measurement property	Raters and testing schedule	Participants	Muscles tested	Statistical measures	Results
Chiu and Lo [4]	Reliability (test-retest)	Raters not reported Between day testing Time interval: 2–3 days	CNP	Neck flexor, extensor, left and right lateral flexors, protractors, and retractors	ICC (95% CI) and 2-way nested ANOVA random model	**ICC (95% CI)** Flexion: 0.99 (0.97, 1.0) Extension: 0.95 (0.90, 0.99) Right lateral flexion: 0.92 (0.74, 0.97) Left lateral flexion: 0.94 (0.89, 0.97) Protraction**:** 0.99 (0.97, 1.0) Retraction**:** 0.97 (0.95, 0.99)
Cibulka et al. [3]	Reliability (intra-rater) Measurement error	2 raters Same day testing Time interval: 1 min	CNP	Left and right Sternocleidomastoid	ICC (2.2) (95% CI) MDC_95_ SEM = SD × √ (1-ICC)	**ICC (95% CI)** Left: 0.97 (0.95–0.99) Right: 0.95 (0.1–0.98) **SEM** Left: 1.82 Right: 1.67 **MDC** Left: 3.16 Right: 3.56
Jordan et al. [11]	Reliability (Intra-rater) Measurement error	1 rater Same day testing Time interval: 1-2 minutes	CNP	Neck flexors and extensors	A linear regression model analysis to derive the correlation coefficient (*r*) CoV	**r** Maximal isometric torque in flexion: *r* = 0.968 (*p* > 0.001) Maximal isometric torque in extension: *r* = 0.938 (*p* > 0.001) **CoV** Flexion: 6.5% Extension: 13% No systematic difference between recordings (*p* > 0.05)
O’Leary et al. [25]	Reliability (test-retest) Measurement error	1 rater Between day testing Time interval: 2 weeks	CNP	Cranio-cervical flexors	ICC (2.1) SEM	***(MVIC peak torque):*** **ICC** Inner torque–0.93 Middle torque–0.91 Outer torque–0.87 **SEM (Nm)** Inner torque–0.7 Middle torque–1 Outer torque–1
Pearson et al. [26]	Reliability (test-retest) Measurement error	Raters not reported Between day testing Time interval: 48 h	WAD	Neck flexors, extensors, right and left lateral flexors, protractors, and retractors	ICC (3.3) (95% CI) SEM = SD × √ (1–ICC) MDC (90% CI) = SEM × √2 × (*z* score)_90_ CoV	**ICC (95% CI)** Flexion: 0.95 (0.83–0.98) Extension: 0.98 (0.93–0.99) Protraction: 0.85 (0.54–0.95) Retraction: 0.89 (0.64–0.96) Left lateral flexion: 0.93 (0.77–0.98) Right lateral flexion: 0.87 (0.59–0.96) Composite: 0.95 (0.84–0.98) **SEM (N)** On average, ±9.1 *N* for an individual with WAD Flexion: 7.5 Extension: 6.2 Protraction: 13.7 Retraction: 14.6 Left lateral flexion: 6.2 Right lateral flexion: 6.4 Composite: 9.1 ± 3.9 **MDC (90% CI) (*N*)** Average ± SD MDC_90_ values for force recordings were 21.1 ± 9.2 *N* for the WAD group Flexion: 17.5 Extension: 14.5 Protraction: 31.9 Retraction: 33.9 Left lateral flexion: 14.4 Right lateral flexion: 14.8 Composite: 21.1 ± 9.2 **CoV (%)** Flexion: 18.9 Extension: 11.2 Protraction: 30.9 Retraction: 29.9 Left lateral flexion: 16.2 Right lateral flexion: 18.9 Composite: 21 ± 7.8
Scheuer and Friedrich [28]	Reliability (Intra and inter-rater)	Intra-rater: 1 rater Inter-rater: 2 raters **Intra-rater (short term):** same-day testing Time interval: 30–60 min **(long term):** between-day testing Time interval: 3–5 days **Inter-rater:** same-day testing Time interval: 30–60 min	CNP	Neck flexors, extensors, left and right lateral flexors	ICC	**ICC** Flexion: 0.85 Extension: 0.76 Left lateral flexion: 0.80 Right lateral flexion: 0.87
Shahidi et al. [30]	Reliability (inter-rater) Measurement error	2 raters Between-day testing Time interval: Mean ± SD (range) 9 ± 4 days (3–14 days)	CNP	Neck flexors, extensors and lateral flexors	ICC (2.1) (95% CI) (two-way random effects model for absolute agreement MDC = 1.96 × √2 × SD × √ (1–test-retest reliability coefficient)	**ICC (95% CI)** Flexion: 0.54 (0.05–0.81) Extension: 0.39 (−0.10–0.73) Left side flexion: 0.72 (0.37–0.89) Right side flexion: 0.56 (0.22–0.82) **MDC** Flexion: 8.7 Extension: 12.5 Left side flexion: 6.3 Right side flexion: 7.2
Vernon et al. [33]	Reliability (intra-rater)	Raters not reported Same day testing Time interval: 5 secs	CNP	Neck flexors, extensors, right rotators and right lateral flexors	ICC	**ICC** Flexion: 0.98 Extension: 0.95 Right rotation: 0.99 Right lateral flexion: 0.98 All ranges of motion: 0.98
Ylinen et al. [37]	Reliability (test-retest)	2 raters Between day testing Time interval: 1 day	CNP	Neck flexor, extensor left and right rotators	ICC	**ICC** Flexion: 0.86 (0.70–0.94) Extension: 0.98 (0.94–0.99) Left rotation: 0.75 (0.49–0.89) Right rotation: 0.74 (0.47–0.89)

*ANOVA* analysis of variance, *CI* confidence intervals, *CNP* chronic neck pain, *CoV* coefficient of variance, *ICC* intra-class coefficients; *MDC* minimal detectable change, *MVIC* maximal voluntary isometric contraction, *SDD* smallest detectable difference, *SEM* standard error of measurement, *SRD* smallest real difference, *WAD* Whiplash-associated disorder

### Risk of bias and overall quality of evidence

Table 3 summarizes the risk of bias for individual studies categorised per neck strength outcome measure and measurement property. Overall, the risk of bias was rated as doubtful or inadequate for most reliability studies, with only one study [30] rated as adequate. The study evaluating validity [4] was rated doubtful. The overall quality of evidence was rated low or very low for the measurement properties of all neck strength measures.

**Table 3:** Summary of risk of bias, criteria for good measurement properties, and overall quality of evidence (GRADE)

Measurement property outcome measure	Study	Risk of bias	Criteria for good measurement properties	Overall rating	Quality of evidence
Reliability					
**Handheld dynamometer**	Cibulka et al. [3]	Doubtful	+	+	Very Low
Intra-rater					
**Handheld dynamometer**	Shahidi et al. [30]	Adequate	-	-	Very Low
Inter-rater					
**Isometric dynamometer**	O’Leary et al. [25]	Inadequate	+	+	Very Low
Test-retest					
**Strain gauge dynamometer**	Jordan et al. [11]	Inadequate	?	?	Low
Intra-rater	Ylinen et al. [37]	Doubtful	?		
**Modified sphygmomanometer dynamometer**	Vernon et al. [33]	Inadequate	?	?	Very Low
Intra-rater					
**Multi-cervical unit**	Chiu and Lo [4]	Doubtful	+	+	Low
Test-retest	Pearson et al. [26]	Doubtful	+		
**Multifunctional measurement unit**	Scheuer and Friedrich [28]	Doubtful	+	+	Very Low
Intra-rater					
**Multifunctional measurement unit**	Scheuer and Friedrich [28]	Doubtful	+	+	Very Low
Inter-rater					
Measurement error					
**Handheld dynamometer**	Cibulka et al. [3]	Doubtful	?	?	Moderate
	Shahidi et al. [30]	Adequate	?		
**Isokinetic dynamometer**	Cagnie et al. [2]	Doubtful	?	?	Very Low
**Isometric dynamometer**	O’Leary et al. [25]	Inadequate	?	?	Low
**Strain gauge dynamometer**	Jordan et al. [11]	Doubtful	?	?	Low
**Multi-cervical unit**	Pearson et al. [26]	Doubtful	?	?	Very Low
Construct validity	Chiu and Lo [4]	Doubtful	?	?	Very Low

Key: Sufficient (+), insufficient (−), Indeterminate (?)

### Synthesis of results

#### Validity

None of the studies included in this review evaluated content validity or criterion validity, with just one study focused on construct validity [4]. Due to the absence of “gold standard” in measuring isometric neck strength, direct comparison was not applicable to establish validity. Instead, a method of contrast group comparison was used to compare mean isometric neck strength between people with and without neck pain. The risk of bias was rated as doubtful and indeterminate for the COSMIN good criteria for good measurement properties. Overall, this study yielded very low quality of evidence for the construct validity of isometric neck strength.

### Reliability and measurement error

#### Handheld dynamometer

One study evaluated intra-rater [3], and one evaluated inter-rater reliability of HHD [30]. Cibulka et al. [3] used a Microfet HHD (Hogan Health Industries, UT, USA), while Shahidi et al. [30] used a FPIX HHD (100kg load cell, Wagner Instruments, CT, USA) for testing. One reported excellent intra-rater reliability [3], and the other reported acceptable inter-rater agreement across time [30]. The risk of bias was rated doubtful [3] and adequate [30] for intra- and inter-rater reliability, respectively. The intra-rater reliability study rated sufficient [3] on the COSMIN criteria while the other study was rated insufficient for inter-rater reliability [30]. Very low overall quality for both intra- and inter-rater reliability for HHD indicates very limited confidence in the reliability estimate within CNP population.

The same studies investigated measurement error [3, 30], with results summarized in Table 2. For the risk of bias, one study was rated doubtful [3] and the other study was rated adequate [30]. Both studies were rated as indeterminate for the COSMIN criteria for good measurement properties. Moderate overall quality indicates moderate confidence in measurement error estimates of HHD to measure neck strength of people with CNP.

#### Isometric dynamometer

One study evaluated test-retest reliability of isometric dynamometer [25] using a NeckMetrix dynamometer (UniQuest Pty Ltd., The University of Queensland, Australia) with overall conclusions reported as good reliability over two sessions of maximal voluntary isometric contraction measurement. The risk of bias was rated inadequate, and test-retest rated as sufficient on the COSMIN criteria for good measurement properties. Overall, very low-quality evidence indicates very little confidence in the reliability estimate of isometric dynamometer within the CNP population.

Measurement error was evaluated in the same study [25]. The risk of bias was rated as inadequate, with the COSMIN criteria rated as indeterminate. The overall low quality indicates little confidence in the measurement error of isometric dynamometer within the CNP population.

#### Strain gauge dynamometer

Two studies evaluated intra-rater reliability of SGD (Neck Exercise Unit, Follo, Norway [11];), the other study used a neck strength measurement system with 2 parts having strain gauges of their own (Kuntovaline Inc, Helsinki, Finland [37];), both studies reported good reliability, with ICCs ranging from 0.74 to 0.96 [37] and correlation coefficient ranging from 0.938 to 0.968 [11]. The risk of bias was rated inadequate [11] and doubtful [37]. Both studies were rated indeterminate on the COSMIN criteria for good measurement properties. Low overall quality indicates limited confidence in the reliability estimates of SGD within the CNP population.

Measurement error was investigated in one study [11]. The risk of bias was rated as doubtful and indeterminate on the COSMIN criteria for good measurement properties. Low overall quality indicates little confidence in the measurement error of SGD within the CNP population.

#### Modified sphygmomanometer dynamometer

One study evaluated intra-rater reliability of MSD using a Comparative Muscle Tester (Magnatec Co. Ltd., Ontario, Canada). Overall conclusions reported high level of accuracy, performance-related reliability, and consistency [33]. The risk of bias was rated inadequate with a rating of indeterminate on the COSMIN criteria for good measurement properties. Very low quality for intra-rater reliability of MSD indicates very limited confidence in the reliability estimate within the CNP population. No studies were identified for measurement error with this outcome measure.

#### Multi-cervical unit

Two studies evaluated test-retest reliability the MCU, both reporting good to excellent reliability (MCU, BTE Technologies, Inc., [26]; Hanoun Medical Inc., Ontario [4];). The risk of bias was rated as doubtful for both studies, and both rated sufficient for the COSMIN criteria for good measurement properties. Low overall quality test-retest reliability for MCU indicates limited confidence in the reliability estimate within the chronic neck pain population.

One study investigated measurement error with results summarized in Table 2 [26]. The risk of bias was rated as doubtful, with the COSMIN criteria for good measurement properties rated as indeterminate. The very low overall quality indicates very little confidence in measurement error estimate for the MCU within a CNP population.

#### Multifunctional measurement unit

One study evaluated intra- and inter-rater reliability of multifunctional measurement unit using Back Check 607 [28]. Overall conclusions were reported as excellent intra- and inter-rater reliability. The risk of bias was rated as doubtful and a rating of sufficient for COSMIN criteria for good measurement properties. Very low overall quality for both intra- and inter-rater reliability indicates little to very little confidence in the reliability estimates for multifunctional measurement unit within the CNP population. No studies were identified for measurement error with this outcome measure.

#### Responsiveness

No studies were identified which evaluated responsiveness.

## Discussion

This systematic review, which evaluated outcome measures of neck strength and their measurement properties in people with CNP, identified six measures used to evaluate neck strength, with the majority of the research investigating people with CNP of non-traumatic origin. The variety of outcome measures found to assess neck strength demonstrates the lack of agreement and gold standard regarding the most appropriate measure for neck strength. To ensure comprehensiveness, all available measures were included in this review. Nevertheless, our review revealed that a consensus on the most optimal outcome measure is still needed to facilitate future research for greater standardisation of neck muscle strength measures across studies.

Reliability was evaluated for all six measures; measurement error was evaluated for the HHD, isokinetic, and isometric dynamometers, SGD and MCU; and validity was evaluated only for the MCU, but no study evaluated responsiveness. The risk of bias for all studies was rated as doubtful or inadequate apart from the study which investigated inter-rater reliability and measurement error of a HHD, which was rated as adequate [30]. For reliability, the overall quality was rated as very low for all outcome measures aside from SGD and MCU which was rated low. All these studies contained small sample sizes with poor overall methodological quality, hence contributing to the high risk of bias and low overall quality for reliability. For measurement error, the HHD was rated moderate for overall quality of evidence, whilst isometric dynamometry and SGD were rated as low. The isokinetic dynamometer and MCU were rated very low for overall quality. For the validity, the quality of evidence was rated very low due to imprecision, as the total sample size of the study was less than 50.

Several factors in the reliability studies included in this review contributed to the high risk of bias score and low or very low overall quality of evidence for each measure. Besides impreciseness, the quality of the methodology in many studies was varied as information regarding the study design was lacking, particularly in the description of experimental preparation, examiners/raters’ positions, and their expertise or training using the measurement tool. Two important aspects of internal validity, randomization and blinding of raters, were also poorly documented across studies. Both elements of the study design are fundamental methodological features in avoiding selection bias and insuring against accidental bias [31]. The reported time interval between measurements were inconsistent amongst studies, varying from seconds to weeks. According to COSMIN, 2 weeks are the recommended time interval for PROM measurements [19, 20]. However, in the context of evaluating neck muscle strength, a period of 2 weeks [25] could be argued to be too long, as it provides time for changes in neck muscle strength to potentially take place. On the other hand, an interval of 1 min [3] is likely to allow recall bias to occur in participants due to a lack of a washout period. Establishment of consensus on a standardized time interval is warranted to minimize measurement variations and improve methodological quality of future studies. Furthermore, variations in muscle testing protocols were observed across studies, which potentially influence the reliability or validity of each neck muscle strength measure, making it difficult to establish the most appropriate neck muscle strength outcome measure without consistent measurement procedures.

Another issue found within the studies is the obscurity around statistical measures used to evaluate the reliability and measurement error of measures. Some studies did not describe the model or formula used for statistical analysis of data. COSMIN recommends the intraclass correlation coefficient as the preferred statistical method for continuous scores in evaluating reliability [19, 20]; however, this was not carried out in one study [11].

### Methodological considerations

Some limitations of the present review are recognised and should be mentioned. Only articles that were published in English were included. Moreover, as the results were found to be heterogeneous, a meta-analysis was not applicable. Instead, a narrative synthesis was conducted to recapitulate the findings. Based on the low quality of the studies included, firm conclusions or recommendations could not be made regarding the most appropriate neck muscle strength outcome measures to use to evaluate neck strength and monitor changes in patients with CNP.

### Implications

The findings from this systematic review have the following future implications for research and clinical practice:

1. A range of outcome measures are used to examine neck muscle strength and as such, there remains a lack of consensus and standardized approach in performing neck strength measurements.
2. This review unveiled methodological flaws in existing studies evaluating measurement properties of neck strength measures. Future research should carefully consider study design and reporting of results (e.g., better description of examiners, adequate time between measurements, reporting of blinding of examination, outlining statistical model for data analysis, etc.) in order to ensure future results with higher overall quality of evidence.

## Conclusion

This systematic review examined the measurement properties of six outcome measures used to evaluate neck muscle strength in people with CNP. Apart from one study evaluating reliability and measurement error, the risk of bias for all studies was rated as doubtful or inadequate. The overall quality of evidence for all measurement properties was rated as low or very low, apart from measurement error of a handheld dynamometer. Due to variability in methodologies and statistical methods, it was difficult to establish the reliability of various neck strength measures, in order to recommend an optimal outcome measure to evaluate neck muscle strength in people with CNP. Further high-quality research is required to evaluate measurement properties of neck muscle strength measures in order to determine the most appropriate measure for future use.

## Supplementary Information

**Additional file 1: Appendix 1.** Search strategy. **Appendix 2.** Summary of data extracted from included studies. **Appendix 3.** Measurement procedures for included studies.
