# Supplementary material for: Measures of neck muscle strength and their measurement properties in adults with chronic neck pain—a systematic review
Source: Syst Rev. 2023 Jan 16;12:6. doi: 10.1186/s13643-022-02162-5 (PMC9841635; doi:10.1186/s13643-022-02162-5)
Supplement: Supplementary file 1 — Additional file 1: Appendix 1. Search strategy. Appendix 2. Summary of data extracted from included studies. Appendix 3. Measurement procedures for included studies. [file 13643_2022_2162_MOESM1_ESM.docx]

**Appendix**

***Appendix 1: Search Strategy***

**EBSCOHost SPORTDiscuss DATABASE SEARCH**

| # | Searches | Results |
| --- | --- | --- |
| 1 | (MH “Neck Muscles”) OR (MH “Neck Muscles/PP”) OR neck OR cervical OR “cervical spine muscle*” OR “cervical muscle*” | 15,916 |
| 2 | (MH “Muscle Strength”) OR (MH "Muscle Strength/PH") OR (MH “Isometric Contraction”) OR “neck muscle strength” OR “neck muscle force” OR “neck force” OR “cervical strength” OR cervical-strength OR “neck strength” OR neck-strength OR “muscle strength testing” OR “isometric neck strength” OR “isometric strength” OR (MH “Muscle Strength Dynamometer”) OR “neck muscle torque” OR “cervical muscle torque” OR “neck MVC” OR “cervical MVC” OR “neck max* contract*” OR “cervical max* contract*” OR “neck manual muscle testing” OR “cervical manual muscle testing” | 3,686 |
| 3 | (MH “Reproducibility of results”) OR “test-retest reliability” OR “intrarater reliability” OR “intra-rater reliability” OR “intratester reliability” OR “intra-tester reliability” OR “interrater reliability” OR “inter-rater reliability” OR “intertester reliability” OR “inter-tester reliability” OR reliability OR reliable OR reproducibility OR reproducible OR validity OR validation OR valid OR responsiveness OR responsive OR clinimetric* OR clinometric* OR psychometric* | 56,651 |
| 4 | (MH “Adult”) OR “healthy adult*” OR “normal adult*” OR “asymptomatic adult*” OR “symptomatic adult*” OR (MH "Neck Pain/PP") OR (MH "Neck Pain/RH") OR “neck pain*” OR “cervical pain*” OR ”neck ache*” OR “chronic neck pain” OR “cnp” OR “cervicogenic” OR “cervicalgia*” OR “cervicodynia*” | 6,211 |
| 5 | 1 and 2 and 3 and 4 | 15 |

**EBSCOHost CINAHL PLUS DATABASE SEARCH**

| # | Searches | Results |
| --- | --- | --- |
| 1 | (MH “Neck Muscles”) OR (MH “Neck Muscles/PP”) OR neck OR cervical OR “cervical spine muscle*” OR “cervical muscle*” | 112,233 |
| 2 | (MH “Muscle Strength”) OR (MH "Muscle Strength/PH") OR (MH “Isometric Contraction”) OR “neck muscle strength” OR “neck muscle force” OR “neck force” OR “cervical strength” OR cervical-strength OR “neck strength” OR neck-strength OR “muscle strength testing” OR “isometric neck strength” OR “isometric strength” OR (MH “Muscle Strength Dynamometer”) OR “neck muscle torque” OR “cervical muscle torque” OR “neck MVC” OR “cervical MVC” OR “neck max* contract*” OR “cervical max* contract*” OR “neck manual muscle testing” OR “cervical manual muscle testing” | 22,909 |
| 3 | (MH “Reproducibility of results”) OR “test-retest reliability” OR “intrarater reliability” OR “intra-rater reliability” OR “intratester reliability” OR “intra-tester reliability” OR “interrater reliability” OR “inter-rater reliability” OR “intertester reliability” OR “inter-tester reliability” OR reliability OR reliable OR reproducibility OR reproducible OR validity OR validation OR valid OR responsiveness OR responsive OR clinimetric* OR clinometric* OR psychometric* | 569,639 |
| 4 | (MH “Adult”) OR “healthy adult*” OR “normal adult*” OR “asymptomatic adult*” OR “symptomatic adult*” OR (MH "Neck Pain/PP") OR (MH "Neck Pain/RH") OR “neck pain*” OR “cervical pain*” OR ”neck ache*” OR “chronic neck pain” OR “cnp” OR “cervicogenic” OR “cervicalgia*” OR “cervicodynia*” | 1,173,516 |
| 5 | 1 and 2 and 3 and 4 | 112 |

**OVID MEDLINE DATABASE SEARCH**

| # | Searches | Results |
| --- | --- | --- |
| 1 | Neck Muscles/ or Neck Muscles/pp or neck.mp. or cervical.mp. or cervical spine muscle*.mp. or cervical muscle*.mp. | 437,433 |
| 2 | Muscle Strength/ or Muscle Strength/ph or Isometric Contraction/ or neck muscle strength.mp. or neck muscle force.mp. or neck force.mp. or cervical strength.mp. or cervical-strength.mp. or neck strength.mp. or neck-strength.mp. or muscle strength testing.mp. or isometric neck strength.mp. or isometric strength.mp. or Muscle Strength Dynamometer/ or neck muscle torque.mp. or cervical muscle torque.mp. or neck MVC.mp. or cervical MVC.mp or neck max* contract*.mp. or cervical max* contract*.mp. or neck manual muscle testing.mp. or cervical manual muscle testing.mp. | 36,038 |
| 3 | Reproducibility of results/ or test-retest reliability.mp. or intrarater reliability.mp. or intra-rater reliability.mp. or intratester reliability.mp. or intra-tester reliability.mp. or interrater reliability.mp. or inter-rater reliability.mp. or intertester reliability.mp. or inter-tester reliability.mp. or reliability.mp. or reliable.mp. or reproducibility.mp. or reproducible.mp. or validity.mp. or validation.mp. or valid.mp. or responsiveness.mp. or responsive.mp. or clinimetric*.mp. or clinometric*.mp. or psychometric*.mp. | 1,264,402 |
| 4 | Adult/ or healthy adult*.mp. or normal adult*.mp. or asymptomatic adult*.mp. or symptomatic adult*.mp. or Neck Pain/pp or Neck Pain/rh or neck pain*.mp. or cervical pain*.mp. or neck ache*.mp. or chronic neck pain.mp. or cnp.mp. or cervicogenic.mp. or cervicalgia*.mp. or cervicodynia*.mp. | 5,109,015 |
| 5 | 1 and 2 and 3 and 4 | 111 |

**OVID EMBASE DATABASE SEARCH**

| # | Searches | Results |
| --- | --- | --- |
| 1 | Neck Muscles/ or neck.mp. or cervical.mp. or cervical spine muscle*.mp. or cervical muscle*.mp. | 649,605 |
| 2 | Muscle Strength/ or Isometric Contraction/ or neck muscle strength.mp. or neck muscle force.mp. or neck force.mp. or cervical strength.mp. or cervical-strength.mp. or neck strength.mp. or neck-strength.mp. or muscle strength testing.mp. or isometric neck strength.mp. or isometric strength.mp. or Muscle Strength Dynamometer/ or neck muscle torque.mp. or cervical muscle torque.mp. or neck MVC.mp. or cervical MVC.mp or neck max* contract*.mp. or cervical max* contract*.mp. or neck manual muscle testing.mp. or cervical manual muscle testing.mp. | 84,356 |
| 3 | Reproducibility of results/ or test-retest reliability.mp. or intrarater reliability.mp. or intra-rater reliability.mp. or intratester reliability.mp. or intra-tester reliability.mp. or interrater reliability.mp. or inter-rater reliability.mp. or intertester reliability.mp. or inter-tester reliability.mp. or reliability.mp. or reliable.mp. or reproducibility.mp. or reproducible.mp. or validity.mp. or validation.mp. or valid.mp. or responsiveness.mp. or responsive.mp. or clinimetric*.mp. or clinometric*.mp. or psychometric*.mp. | 1,775,554 |
| 4 | Adult/ or healthy adult*.mp. or normal adult*.mp. or asymptomatic adult*.mp. or symptomatic adult*.mp. or Neck Pain/rh or neck pain*.mp. or cervical pain*.mp. or neck ache*.mp. or chronic neck pain.mp. or cnp.mp. or cervicogenic.mp. or cervicalgia*.mp. or cervicodynia*.mp. | 7,578,131 |
| 5 | 1 and 2 and 3 and 4 | 197 |

**WEB OF SCIENCE DATABASE SEARCH**

| # | Searches | Results |
| --- | --- | --- |
| 1 | ts=(cervical) OR ts=(cervical spine muscle*) or ts=(cervical muscle*) | 228,012 |
| 2 | ts=(Muscle Strength) OR ts=(Isometric Contraction) OR ts=(cervical muscle strength) OR ts=(cervical muscle force) OR ts=(cervical strength) OR ts=(cervical-strength) OR ts=(muscle strength testing) OR ts=(isometric strength) OR ts=(Muscle Strength Dynamometer) OR ts=(cervical muscle torque) OR ts=(cervical MVC) OR ts=(cervical max* contract*) OR ts=(cervical manual muscle testing) | 84,753 |
| 3 | ts=(Reproducibility of results) OR ts=(test-retest reliability) OR ts=(intrarater reliability) OR ts=(intra-rater reliability) OR ts=(intratester reliability) OR ts=(intra-tester reliability) OR ts=(interrater reliability) OR ts=(inter-rater reliability) OR ts=(intertester reliability) OR ts=(inter-tester reliability) OR ts=(reliability) OR ts=(reliable) OR ts=(reproducibility) OR ts=(reproducible) OR ts=(validity) OR ts=(validation) OR ts=(valid) OR ts=(responsiveness) OR ts=(responsive) OR ts=(clinimetric*) OR ts=(clinometric*) OR ts=(psychometric*) | 2,684,014 |
| 4 | ts=(Adult) OR ts=(healthy adult*) OR ts=(normal adult*) OR ts=(asymptomatic adult*) OR ts=(symptomatic adult*) OR ts=(cervical pain) OR ts=(cervicogenic) OR ts=(cervicalgia*) OR ts=(cervicodynia*) | 1,638,398 |
| 5 | 1 and 2 and 3 and 4 | 359 |

***Appendix 2: Summary of data extracted from included studies***

| **Content** | **Data items** |
| --- | --- |
| Bibliographic data | Authors, year of publication |
| Study characteristics | Study design, sample size |
| Setting | Country, setting of measurement |
| Participants characteristics | Age, gender, healthy or with neck pain (disability, pain intensity, duration of pain etc.) |
| Outcome measures | NS measures.  Type of muscle contraction measured.  Measurement procedure: warm up, participants’ position, fixation, examiners’ position (if applicable), line of force (resistance), number of repetitions, duration of rest etc. |
| Measurement properties | Measurement properties (reliability, validity and responsiveness), statistical methods used and results. |

***Appendix 3: Measurement procedures for included studies***

Chiu and Lo (2002)

**Warm up/preparation:** The first session was used as a trial run so that the subjects could become familiar with the procedure and the machine. Both the seat height and the position of the armrest were recorded to ensure a standardized position for repeated testing.

**Participants’ Positioning:** Subjects sat in the adjustable chair

**Examiners' position:** Not mentioned

**Procedure in brief:** The subject was instructed to do three consecutive steady contractions (in different directions for testing different muscle groups) as hard as possible

**Fixation:** During the measurement, the subject again sat in the adjustable chair with his/her trunk secured by the shoulder restraint system. The inner head brace was secured comfortably around the head of the subject.

**Line of force (resistance):**  A load cell fitted into the brace was used to measure the isometric force applied by the subject for the six directions. Examiner placed a hand-held dynamometer over the left temple. Subject was instructed to raise the head off table surface by maximally pressing against the dynamometer

**Number of repetitions:** Three measurements were taken for each of the six directions randomly (flexion, extension, lateral flexions, protraction and retraction) at each session.

**Duration of rest:** 10 seconds rest between each contraction and 2 minutes rest between different directions to avoid fatigue within a session.

**Recordings of measurement:** The load cell was connected to the MRC unit with an objective documentation and evaluation system through a direct system interface. The system’s software automatically recorded (sampling rate 20/second) and calculated the average and peak isometric strength (PIS) for six different directions among the three trials.

**Reliability testing:** Data collected from the second and third sessions were used for analysis

**Blinding of Raters** – not reported

**Randomisation** – measurements were taken for 6 directions randomly

Cibulka et al. (2017)

**Warm up/preparation:** Not mentioned

**Participants' position:** Subject in supine and the head rotated opposite of the muscle being tested.

**Examiners' position:** Not mentioned

**Procedure brief:** The subject was instructed to raise their head with the dynamometer held on their temple, with the muscle tester not allowing any neck motion, thereby creating a three second isometric contraction in the form of a “make” test, where the tester did not try to “break” the isometric muscle contraction but asked the subject to maximally push into the HHD.

**Fixation:** Not reported

**Line of force (resistance):**  The examiner placed a hand-held dynamometer over the left temple. The subject was instructed to raise the head off the surface of the table by maximally pressing against the dynamometer

**Number of repetitions:** 3 for each side

**Duration of rest:** Alternating the test sides allowed subjects a one-minute rest break between trials.

**Unit of measurement:** This measurement was then recorded in kilograms (kg) by another researcher.

**Reliability testing:** The last two measures taken were used for reliability data analysis, while the mean of the three measures was used for group analysis.

**Randomisation** – first side to be measured was randomly determined by flipping of coin

**Blinding of Raters** – Two other DPT students took the measurements from the two assessors.

Jordan et al. (1997)

**Warm up/preparation:** The measuring device as recalibrated before each measuring session with the aid of a known 10-kg weight. Before testing, all patients were demonstrated by the principal investigator. Patients were then able to "practice" with light resistance (2-3 kg in flexion, 3-4 kg in extension: 5-6 repetitions in each direction).

**Participants’ position:** Flexion was measured at 45° from the neutral position, and extension at 30°.

**Fixation:** To minimize the effects of limb movements on CC flexor muscle performance, the subject’s legs were suspended on slings so that the knees and the hips were flexed to 45 degrees and the arms were folded across the chest. Soft straps were attached to the supporting surface and were secured lightly over the subject’s shoulders to avoid movement of the trunk on the supporting surface.

**Line of force (resistance):** Not mentioned – axis of motion was joint between the C7 and T1.

**Number of repetitions:** 2 maximal isometric strength tests were made for both the flexors and the extensors of the cervical spine. A third measurement was taken if there was >25% difference between the first two measurements.

**Duration of rest:** 1-2 min rest between measurements

**Randomisation** – Not reported

**Blinding of Raters** – No

O’Leary et al. (2005)

**Warm up/preparation:** All subjects were given standard instructions, familiarization of the testing procedure, and a standard warm-up in all 3 ranges immediately before the trial in that range. Subjects were instructed to nod their head so that their mandible pushed downward on the application pad of the dynamometer to elevate the visual display column maximally. They practiced performing the task, ensuring that the head remained in contact with the head platform and that the teeth remained occluded to minimize the potential contribution of the mandibular depressors. Warm-up consisted of 4 submaximal repetitions, with each successive repetition at a greater intensity than the previous one, and a fifth repetition to their maximal ability.

**Participants’ position:** All tests were performed with the subject in a supine position. For inner-range measurements, the subject’s head and lever arm was positioned in 10 degrees of head flexion from the neutral position. For outer-range measurements, the subject’s head and lever arm was positioned in 10 degrees of head extension from the neutral position.

**Examiners’ position –** Not reported

**Procedure brief:** In each session, MVIC recordings were made in the inner, middle, and outer ranges of CC flexion. The MVIC recordings were always completed first, and the order of testing was randomized among subjects but was consistent within subjects and between sessions. Subjects were instructed to completely relax between repetitions, ensuring that no active force was placed on the application pad of the dynamometer until commencement of the next trial.

**Fixation:** To minimize the effects of limb movements on CC flexor muscle performance, the subject’s legs were suspended on slings so that the knees and the hips were flexed to 45 degrees and the arms were folded across the chest. Soft straps were attached to the supporting surface and were secured lightly over the subject’s shoulders to avoid movement of the trunk on the supporting surface.

**Line of force (resistance):** The subject’s AOR landmark (concha of the ear) and the dynamometer axis were aligned with the head in a neutral CC flexion/extension position according to a standard anthropometric neutral position of the head (Frankfort plane). In this craniocervical position, with the subject positioned supine, a vertical line bisects the orbitale and the tragion anatomical landmarks to position the craniocervical spine in a neutral flexion/ extension position. The dynamometer-subject axes were aligned with the aid of a Web camera (QuickCam Pro 4000) erected perpendicular to the axis of the dynamometer.

**Number of repetitions:** Three MVIC trials per range were performed

**Duration of rest:** 60 seconds of rest between maximal efforts for a range. 5 minutes of rest between ranges.

**Duration of contraction:** Each contraction lasted between 3 and 5 seconds.

**Randomisation** – Not reported

**Blinding of Raters** – No

Pearson et al. (2009)

**Warm up/ preparation:** Three submaximal practice trials were performed for each direction prior to recording. Examiners’ guidance and correction was provided as needed during these practice trials.

**Participants’ positioning:** Subjects were seated in the MCU chair and a proper position of the cervical spine in neutral was obtained by adjusting the height and position of the seat and back of the chair. The headpiece was also adjusted and positioned 15° below the horizontal for flexion, extension, protraction, and retraction testing, and 0° from the horizontal for both lateral flexions. For lateral flexion testing, the headpiece was also rotated by 90°. Pads were positioned just above the upper portion of the eyebrows for flexion and protraction testing, just above the external occipital protuberance for extension and retraction testing, and just above the upper part of the earlobes for lateral flexion testing.

**Procedure in brief:** During the testing phase, each subject was asked to perform 3 consecutive trials of 3 seconds each of MVIF in each direction. For each set of 3 trials, subjects were instructed to gradually increase the force to their maximum. Instructions to the subjects were as follows: “Push as hard as you can into the pads…by bringing your chin to your chest [flexion]…by bringing the back of the head to the neck [extension]…by bringing your right (left) ear to your right (left) shoulder [lateral flexion]…by bringing your chin forward as much as possible [protraction]…by bringing your chin as far back as possible [retraction].”

**Fixation:** Shoulder and waist straps were adjusted to further isolate the cervical spine from the rest of the body. Subjects were asked to keep their arms crossed with their hands resting on opposite shoulders and their feet crossed on the platform throughout all trials.

**Line of force (resistance):** Not reported

**Number of repetitions:**  3 consecutive trials of 3 seconds each of MVIF in each direction

**Duration of rest**: A 1-minute rest between each trial and a 2-minute rest between each direction.

**Blinding to patients' condition:** Testers were not blinded to the subject’s group assignment.

**Randomisation of order:** The directions were assigned in a random order, and the subjects always began and remained with the cervical spine in the neutral position.

**Recordings of measurement:** For each subject, the mean of the peak values obtained for 3 MVIF trials were calculated for each direction and used for further analysis. Group flexion-extension ratios were computed by averaging the ratios obtained for all subjects at the first experimental session. In addition, percentages of force deficits were calculated for each direction using the formula: [(mean force healthy – mean force WAD)/(mean force healthy)] x 100%.

**Reliability testing:** The protocol was repeated in a second session (day 2) using the same sequence of randomization for directions in order to assess the reliability measurements made with the MCU and to calculate the standard error of measurements (SEM) and the minimal detectable change (MDC) values for each group. Subjects were only allowed to view their force results at the end of the second testing session to avoid bias.

**Randomisation** – Not reported

**Blinding of Raters** – No

Scheuer and Friedrich (2010)

**Warm up/preparation**: 2 measurements of 30% to 50% strength were performed before measuring the maximum muscle strength, which allows the required muscle groups to warm up.

**Participant’s positioning:** Not reported

**Fixation**: subject was fastened into the apparatus at scapula level, with the measuring mechanism positioned just above eye level

**Line of force (resistance)**: Not mentioned

**Number of repetitions**: On each of 2 separate days within a 5-day span, 2 measurements.

**Duration of rest**: 30-60 mins

**Reliability testing**: The first 3 measurements were taken by the same male investigator, while the last was conducted by a female investigator. The second investigator was chosen to be female because we assumed this could affect probands’ (male and female) behavior and ambition doing strength measurements more than another male investigator. Thus, we assessed short-term, long-term, and interrater reliability: short-term comparing t1 and t2, long-term comparing t1 and t3, and interrater comparing t3 and t4.

Shahidi et al. (2012)

**Warm up/preparation:** Both therapists participated in three, one hour training sessions to standardize the examination procedures.

**Participants’ positioning:**

Flexion: Participants were positioned in supine and asked to hold their head in approximately 30 degrees of flexion with the chin tucked.
Extension: Participants were positioned in prone with the shoulders supported at the edge of the examination table and the head held against gravity just beyond the edge of the table. Participants were asked to hold their head in a neutral position.

Side flexion: This outcome was assessed in supine. Participants were instructed to maintain a neutral head position with the back of the head resting against the examination table.

**Examiners' position:** Not mentioned

**Fixation:** Participants were stabilized in supine using a 4-inch Velcro strap placed across the chest at the level of the sixth thoracic vertebrae (T6) and across the pelvis at the level of the anterior superior iliac spine to prevent movement of the upper body as force was applied to the head.

**Line of force (resistance):** For all isometric force measurements, manual resistance was applied at a rate of approximately 3 kg∙F/s and the maximum force recorded by the dynamometer while the participant was still able to maintain the test position was considered the maximum isometric force.
Flexion: The examiner applied a force into the direction of cervical extension with the HHD centered on the forehead.

Extension: The examiner provided a force into the direction of cervical flexion with the HHD centered on the back of the head.

Side flexion: The examiner provided a force into the direction of side bending with the HHD centered on the contralateral side of the head.

**Number of repetitions:** Only one maximum strength test was performed in each direction to minimize the potential for reduced force output with increased cervical pain due to repeated testing

**Duration of rest:** 30-60s break between measurements

**Reliability testing:** Single trial results were used to assess the interrater reliability of isometric cervical strength measurements between sessions.

**Blinding of Rater**: As a safety precaution, therapists were not blinded to group assignment so that any worsening of symptoms could be monitored during the examination. However, all participants were instructed not to provide any clinical information or cues not related to the examination with the therapist prior to each session. Therapists remained blinded to the other rater's findings between examinations.

**Randomisation:** Tests were performed in the same order for each rater

Vernon et al. (1992)

**Warm up/preparation:** Trial runs of each test procedure were conducted in order to ensure proper positioning of the equipment, to confirm proper performance by the subject and to reduce any test apprehension (i.e., to give the subject the feel of the equipment and the test performance).

**Participants’ positioning:** The subject remained standing throughout the test procedure. The air bladder platform was moved to a position such that its top edge was level with the subject's eyebrows. The subject was guided into a position with his feet lined up with a floor marker directly under the platform. In each of the test positions, care was taken to position the subject so that the subject's head rested in the neutral position against the surface of the bladder. As such, a slight preload of the bladder involved no more than 10 degrees of rotation of the head in any plane.

**Fixation:** The assessor maintained gentle manual stabilisation of the subject's pelvis during the test. This guarded against inward drift, which would increase the leverage of the trunk muscles in any of the test positions.

A standardised position of the subject's arms was adopted for all tests such that the arms were raised to the 90 degree/90 degree position.

**Line of force (resistance):** Subjects were instructed to commence, upon verbal signal, a gradual sustained pressure on the air bladder

**Number of repetitions:** 2 trials per group

**Duration of rest:** 5-sec rest interval

**Procedure in brief:** The instrument is programmed with a 2-sec delay such that data are acquired over the last 3 sec of sustained maximal effort. During the resisted test, the assessor gave verbal feedback to the subject instructing them to "keep pushing" or "push as hard as you can" until the buzzer sounded.

**F:** Assessor maintained gentle manual stabilisation of the subject's pelvis during the test. This guarded against inward drift, which would increase the leverage of the trunk muscles in any of the test positions.

A standardised position of the subject's arms was adopted for all tests such that the arms were raised to the 90 degree/90 degree position.

**Modifications for neck pain group:** The protocol of acceptable (but then sustained) maximal effort was used. Subjects were instructed to produce a level of pressure that reached, but did not exceed, tolerable pain. They were instructed to sustain that level of pressure and not to suddenly and prematurely "give up", as that occurrence would be deemed a test failure.

Ylinen et al. (2004)

**Warm up/preparation:** The measurement system was calibrated with standard weights (5kg, 10kg, 15kg, 20kg). Three submaximal warm-up efforts with gradually increasing force were performed in each direction, to acquaint subjects with the testing position and with the neck strength measurement system.

**Participants' positioning:**  Subjects were seated in a standard position. The rotational axis of the upper cervical column, which runs through the center of the dens, was brought into the same line as the shaft of the measurement device. The head was positioned by looking at the opening of both ear canals from 2 sights running parallel to the shaft of the measurement device.

**Fixation:** The head was centralized by a screw system traveling the same distance from both sides and tightening the support against the head.

**Line of force (resistance):**  During the flexion and extension measurements, the subjects pushed directly forward or backward against the padded strain gauge of the neck strength measurement system.

**Number of repetitions:**  Each subject was told to perform at least 3 maximal efforts lasting 5 seconds each. If the third result showed an improvement of more than 5%, additional efforts were performed until the improvement in strength remained under that.

**Duration of rest:** 45-second intervals

**Procedure in brief:** Isometric rotation strength was measured first. Torque was not measured from the head supports but from the shaft of the apparatus to which the head supports were attached. During the flexion and extension measurements, the subjects pushed directly forward or backward against the padded strain gauge of the neck strength measurement system. Verbal encouragement was given in a steady loud voice without shouting. Changing position took about 2 minutes. In the test trials, each subject was told to perform at least 3 maximal efforts lasting 5 seconds each. If the third result showed an improvement of more than 5%, additional efforts were performed until the improvement in strength remained under that. The best result was used in the final analysis.

**Reliability testing:** In the chronic group with neck pain, the strength tests were repeated at the same time on the following day by the same tester, to evaluate repeatability.
